# Spheroid-Derived Cells From Renal Adenocarcinoma Have Low Telomerase Activity and High Stem-Like and Invasive Characteristics

**DOI:** 10.3389/fonc.2019.01302

**Published:** 2019-12-04

**Authors:** Leili Saeednejad Zanjani, Zahra Madjd, Arezoo Rasti, Mojgan Asgari, Maryam Abolhasani, Kevin J. Tam, Raheleh Roudi, Gunhild Mari Mælandsmo, Øystein Fodstad, Yvonne Andersson

**Affiliations:** ^1^Oncopathology Research Center, Iran University of Medical Sciences (IUMS), Tehran, Iran; ^2^Cellular and Molecular Research Center, Iran University of Medical Sciences, Tehran, Iran; ^3^Department of Molecular Medicine, Faculty of Advanced Technologies in Medicine, Iran University of Medical Sciences, Tehran, Iran; ^4^Department of Urologic Sciences, Vancouver Prostate Center, University of British Columbia, Vancouver, BC, Canada; ^5^Department of Basic Sciences/Medical Surgical Nursing, Faculty of Nursing and Midwifery, Tehran University of Medical Sciences, Tehran, Iran; ^6^Hasheminejad Kidney Center, Iran University of Medical Sciences (IUMS), Tehran, Iran; ^7^Department of Tumor Biology, Oslo University Hospital, The Norwegian Radium Hospital, Oslo, Norway

**Keywords:** cancer stem cells, renal cell carcinoma, hTERT, telomerase activity, MST-312

## Abstract

Cancer stem cells (CSCs) are a theorized small subpopulation of cells within tumors thought to be responsible for metastasis, tumor development, disease progression, treatment-resistance, and recurrence. The identification, isolation, and biological characterization of CSCs may therefore facilitate the development of efficient therapeutic strategies targeting CSCs. This study aims to compare the biology and telomerase activity of CSCs to parental cells (PCs) in renal cancer. Renal CSCs were enriched from the ACHN cell line using a sphere culture system. Spheroid-derived cells (SDCs) and their adherent counterparts were compared with respect to their colony and sphere formation, expression of putative CSC markers, tumorigenicity in non-obese diabetic/severe combined immunodeficiency (NOD/SCID) mice, and invasiveness. The expression of genes associated with CSCs, stemness, EMT, apoptosis, and ABC transporters was also compared between the two populations using quantitative real-time PCR (qRT-PCR). Finally, telomerase activity, hTERT expression, and sensitivity to MST-312, a telomerase inhibitor, was investigated between the two populations. We demonstrated that a subpopulation of ACHN cells was capable of growing as spheroids with many properties similar to CSCs, including higher clonogenicity, superior colony- and sphere-forming ability, and stronger tumorigenicity and invasiveness. In addition, SDCs demonstrated a higher expression of markers for CSCs, stemness, EMT, apoptosis, and ABC transporter genes compared to PCs. The expression of hTERT and telomerase activity in SDCs was significantly lower than PCs; however, the SDC population was more sensitive to MST-312 compared to PCs. These findings indicate that the SDC population exhibits stem-like potential and invasive characteristics. Moreover, the reduced expression of hTERT and telomerase activity in SDCs demonstrated that the expressions of hTERT and telomerase activity are not always higher in CSCs. Our results also showed that MST-312 treatment inhibited SDCs more strongly than PCs and may therefore be useful as a complementary targeted therapy against renal CSCs in the future.

## Introduction

Renal cell carcinoma (RCC) is the most lethal neoplasm of all urologic cancers and is responsible for ~90% of renal malignancies and 2–3% of total cancers in adults (1, 2). The incidence of RCC has been increasing worldwide over the past 20 years (1). It is estimated that there will be 65,340 new cases and 14,970 deaths from RCC in the United States in 2018. The 5 year relative survival rate of RCC in the United States was found to be 93% in localized disease, 67% in regional disease, and 12% in patients with metastases (3). Despite improvements in diagnostic techniques, ~25–30% of RCC patients present with metastases at the time of diagnosis. Metastatic RCC is notoriously resistant to most current therapies, and 30% of RCC patients develop recurrent disease after surgery (4, 5).

Accumulating evidence would indicate that a small subpopulation of cells within the tumor, known as cancer stem cells (CSCs), is responsible for metastasis, tumor development, disease progression, treatment resistance, and recurrence in several human cancers, including RCC (4, 6). Therefore, the identification, isolation, and characterization of the molecular and biological features of CSCs will undoubtedly inspire novel therapeutic strategies for patients with cancer.

In 2008, Bussolati et al. first identified a rare subpopulation of cells that exhibit CSC properties in RCCs (7). To date, various strategies have been developed to isolate, enrich, and characterize CSCs including isolation by flow cytometry using specific cell surface markers, detection of side-population (SP) phenotypes by Hoechst 33342 exclusion, evaluation their ability to grow and propagate as spheroids, and assessment of aldehyde dehydrogenase 1 (ALDH1) activity (8). In recent years, the sphere culture system has become one of the most widely used methods to enrich CSCs in human cancers and cancer cell lines, and it overcomes the lack of any pan-cancer universal markers for CSCs (9–11). Zhong et al. enriched CSCs from the SK-RC-42 RCC cell line using this method and characterized their immunophenotype. They demonstrated that sphere-forming cells had many properties similar to CSCs (12). In addition, Lichner et al. enriched and isolated RCC spheroids and showed that they exhibit CSC characteristics, including self-renewal, high tumorigenicity, differentiation capacity, as well as the increased expression of stem cell-related transcription factors and mesenchymal markers (13). At present, few reports exist investigating the biological characteristics of renal CSCs.

Human telomerase, a ribonucleoprotein enzyme, is made up of the catalytic component human telomerase reverse-transcriptase (hTERT), a human telomerase RNA (hTR) molecule, and other associated proteins that protect telomeres from critical shortening, thus allowing continuous cell division (14, 15). Evidence shows that germ cells, stem cells, and tumor cells express significant levels of telomerase in contrast to most normal human somatic cells (16, 17). Several studies have shown that malignant cells from different types of cancer have telomerase expression levels that correlate with tumor-initiating ability (18, 19). However, we could not find any similar data examining the expression levels of hTERT and telomerase activity in renal CSCs. Given the important role of the telomerase and hTERT in stem cells and in the progression of various types of cancer (16, 20), we speculated that telomerase activation plays a critical role in renal CSCs, and that targeting this type of cell may constitute an effective therapeutic strategy in RCC. To test this hypothesis, we generated spheroid-derived cells (SDCs) from renal adenocarcinoma cell lines and evaluated various biological characteristics. We also compared, for the first time, expression levels of hTERT and telomerase activity between parental cells (PCs) and SDCs.

## Materials and Methods

### Cell Lines, Culture, and Sphere Formation

Human RCC cell lines (ACHN, 786-O, and Caki-1) were obtained from the American Type Culture Collection (ATCC). The cell line ACHN was grown in Dulbecco's modified Eagle's medium (DMEM), 786-O in RPMI-1640, and Caki-1 was grown in McCOY's 5A (all, Sigma-Aldrich, USA) supplemented with heat-inactivated (56°C, 30 min) 10% fetal bovine serum (FBS), 2 mM L-glutamine, 2 mM non-essential amino acid, 100 U/ml penicillin, and 100 μg/ml streptomycin (all, Gibco, Invitrogen, USA), also known as a complete medium, in a humidified atmosphere at 37°C under 5% CO_2_. The SDCs were derived by placing the ACHN, 786-O, and Caki-1 cells grown as monolayers into a serum-free medium consisting of 10 ng/ml EGF and 20 ng/ml bFGF (Peprotech, USA) using poly-hydroxyethyl methacrylate (poly-HEMA) (Sigma, USA) coating flasks to prevent cell adhesion as described previously. Approximately 7 × 10^5^ of single viable cells were plated on poly-HEMA coated cell culture flasks in a serum-free medium. Fresh aliquots of EGF and bFGF were added every other day (21). Following culture for 10–12 days, SDCs were collected by gravity, washed with PBS, and detached with accutase (Sigma-Aldrich, USA), and they were then transferred into the cell culture coated flask to promote further generations. The ACHN cell line was selected for this study according to the formation of the SDCs.

### Colony Formation Assay

The single viable cells (100 cells/dish) from PCs and SDCs were seeded into 6-well culture plates (Corning, Costar, USA) containing 2 ml of DMEM with 10% FBS and allowed to grow for 10 days at 37°C. The cell colonies were fixed with 4% paraformaldehyde (Merck, Germany) and stained with 0.05% crystal violet (Sigma, USA). Colonies of each cell population were counted under the microscope (×200) in all fields (21).

### Sphere Formation Assay

The single viable cells (1,000 cells/dish) from PCs and SDCs obtained from the first generation of spheroids were seeded in serum-free medium including 10 ng/ml EGF and 20 ng/ml bFGF, which were added every other day into plates using ultra-low-attachment 6-well plates (Corning, Costar, USA). After 12 days, the spheroid number of each well was counted (22).

### Expression of Putative CSC Markers

Flow cytometry was applied to compare the expression of a panel of CSC markers, some of which are known renal CSC markers, including CD24, CD44, CD133, CD105, CXCR4, and CD73 (23). Other cell surface markers such as CD29, CD34, CD56 (NCAM), CD90, CD117, and CD146 have been found to be differentially expressed between PCs and SDCs of other cancers (24). The SDCs were dissociated by accutase and then washed with PBS. Nearly 1 × 10^5^ single viable cells from each population were incubated with 1 μg/ml fluorescently labeled monoclonal antibodies, or respective isotype controls, in the dark for 30 min on ice. Several antibodies were used: phycoerythrin (PE)-labeled mouse anti-human CD24, PE-labeled mouse anti-human CD29, FITC-labeled mouse anti-human CD34, fluorescein iso thiocyanate (FITC)-labeled mouse anti-human CD44, PE-labeled mouse anti-human CD56, PE-labeled mouse anti-human CD73, FITC-labeled mouse anti-human CD90, PE-labeled mouse anti-human CD105, PE-labeled mouse anti-human CD117, PE-labeled mouse anti-human CD133, PE-labeled mouse anti-human CD146, and PE-labeled mouse anti-human CXCR4 (all, BD Biosciences, USA, except CD90, DAKO, Denmark). Then, the cells were washed, resuspended, and analyzed by flow cytometry (FACS Calibur, BD, USA). The FlowJo Version 7 was also used to analyze the data.

### Cell Invasion Assay

The cell invasion assay was performed by a Cultrex Basement Membrane Extract (BME) Cell Invasion Assay (R&D Systems, USA). Briefly, the PCs and SDCs were dissociated by accutase; single cells were suspended in serum-free DMEM at a concentration of 5 × 10^4^ cells/ml for 18–24 h prior to assay. Then, the medium was removed, and the cells were resuspended at 1 × 10^6^ cells/mL in serum-free medium. The upper chamber was loaded with 50 μl cell suspension, and the lower chamber was loaded with 150 μl DMEM with 5% FBS. The remaining cells were used for the standard curve according to the manufacturer's protocol. Following culture for 48 h, the top and bottom chambers were aspirated and washed. Then Calcein AM solution mixed with cell dissociation solution was added to the bottom chamber and incubated at 37°C for 1 h. The plate was read at 485 nm excitation and 520 nm emission (Bio Tek, USA). The standard curve was used to determine the number of invaded cells as well as the percentage of cell invasion.

### Animals and Xenograft Model for Evaluation of Tumorigenicity

The PCs or SDCs were dissociated, washed twice, and counted. In-house bred, 4- to 5-week-old female non-obese diabetic/severe combined immunodeficiency (NOD-SCID) mice (*n* = 4) were injected subcutaneously on both left and right flank with either 1 × 10^3^, 1 × 10^4^, or 1 × 10^5^ ACHN cells, which were resuspended in 50 μl serum-free medium. The viability of cells was determined using the trypan blue (Sigma-Aldrich, USA) exclusion test. Tumor formation/growth was monitored using hand-held calipers and measured twice weekly. Tumor volume was calculated using the [tumor length × (tumor width^2^)]/2 formula. Eight weeks post-inoculation, the mice were sacrificed by cervical dislocation. Tumor volume was plotted as a function of time (days). Body weight was recorded throughout the experiments. Tumor xenografts were divided in two for RNA isolation and formalin fixation for immunohistochemistry (IHC) (13). All procedures were approved by the National Animal Research Authority and performed according to regulations of the Federation of European Laboratory Animals Science Association.

### RNA Isolation, cDNA Synthesis, and Quantitative Real-Time PCR (qRT-PCR) Analysis of Xenograft Tumors Derived From PCs and SDCs

Frozen xenograft tumor specimens were removed from the freezer and cut into smaller pieces. Total RNA of xenograft tumors from PCs and SDCs were extracted by the Trizol method (Sigma, USA) according to the manufacturer's standard procedures. Following extraction, RNA quantitation was performed using a Nanodrop 2000 spectrophotometer (Thermo Scientific, USA). Complementary DNA (cDNA) was synthesized by q Script™ cDNA Synthesis Kit (Quanta BioSciences, USA) according to the manufacturer's instructions. qRT-PCR was performed to examine the expression levels of a panel of common stemness genes, including OCT4, SOX2, Nanog, and Lin28 and Epithelial–mesenchymal transition (EMT) genes such as Snail1, E-cadherin, Twist1, and Vimentin genes. qRT-PCR was carried out using qScript^TM^ Reverse and qScript^TM^ Reaction (Quanta BioSciences, USA) on a Rotor Gene 6000 Real-Time PCR System (CFX Connect, Bio-Rad, USA) using different programs: 95°C for 3 min, then 39 cycles alternating in turn with 95°C for 15 s, 60°C for 1 s, and 72°C for 1 min, and then maintained at 75°C for 5 min. Comparative gene expression analysis was performed using the Ct method with normalization to the reference gene GAPDH. We tested also RPL32 with various results.

### Immunohistochemistry Staining of Xenograft Tumors Derived From PCs and SDCs

Formalin-fixed xenograft tumor sections derived from PCs and SDCs were first stained with hematoxylin and eosin (H&E) to determine histopathology. IHC was then performed as described before (25, 26) to study protein expression of common stemness genes, including OCT4 and Nanog (R&D Systems, Inc. dilutions 1:50, 1:100, respectively).

### RNA Isolation, cDNA Preparation, and qRT-PCR for Gene Expression Assay

Total RNA was extracted from PCs and SDCs using an RNeasy Mini Kit (Qiagen, USA) according to the manufacturer's protocol (21). cDNA was synthesized using the Reverse Transcription System (Bioneer, South Korea) according to the manufacturer's instructions. The expression level of a panel of genes, including stemness genes (OCT4, SOX2, Nanog, Klf4, C-MYC, Lin28, Nestin, and Rex1) EMT genes (Snail1, Snail2, E-Cadherin, N-Cadherin, Twist1, Twist 2, Zeb1, Zeb2, and Vimentin) apoptosis genes (BCL2, BCL2L12, Fas, Caspase-3, and BAX), ABC transporters genes (ABCG2, ABCB1, and ABCC1), as well as an active component of the telomerase enzyme gene (hTERT), were evaluated in PCs and SDCs using qRT-PCR technique according to the previous mentioned method with normalization to the level of the internal control gene, GAPDH. The sequence-specific primers are shown in Table 1.

**Table 1:** Primers sequences for quantitative real-time PCR (qRT-PCR).

**Genes groups**	**Gene name**	**Primer sequence (5^**′**^ → 3^**′**^)**	**Product size (bp)**
Housekeeping gene	GAPDH	F: CATGAGAAGTATGACAACAGCCT R: AGTCCTTCCACGATACCAAAGT	113
Stemness genes	OCT4	F: GTGGAGAGCAACTCCGATG R: TGCAGAGCTTTGATGTCCTG	121
	SOX2	F: AATGGGAGGGGTGCAAAAGAGG R: GTGAGTGTGGATGGGATTGGTG	143
	Nanog	F: AGCTACAAACAGGTGAAGAC R: GGTGGTAGGAAGAGTAAAGG	145
	Klf4	F: CCTCGCCTTACACATGAAGAG R: CATCGGGAAGACAGTGTGAAA	108
	C-MYC	F: ACACATCAGCACAACTACG R: CGCCTCTTGACATTCTCC	161
	Lin28	F: CTTTGCCTTCGGACTTCTC R: AACTGCTGGTTGGACACG	100
	Nestin	F: AGAGAGCGTAGAGGCAGTAA R: GGTGCTTGAGTTTCTGGAGAT	108
	Rex1	F: TTTACGTTTGGGAGGAGG R: GTGGTCAGCTATTCAGGAG	150
EMT genes	Snail1	F: CCAGAGTTTACCTTCCAGCA R: GATGAGCATTGGCAGCGA	101
	Snail2	F: AACTACAGCGAACTGGACAC R: GGATCTCTGGTTGTGGTATGAC	91
	E-cadherin	F: CAGGAGTCATCAGTGTGGT R: GGAGGATTATCGTTGGTGTCAG	151
	N-cadherin	F: GCCCAAGACAAAGAGACCC R: CTGCTGACTCCTTCACTGAC	94
	Twist1	F: TTCTCGGTCTGGAGGATGGAG R: ACGCCCTGTTTCTTTGAATTTGG	228
	Twist2	F: AGCGACGAGATGGACAATAAG R: TAGTGGGAGGCGGACAT	116
	Zeb1	F: CTTCTCACACTCTGGGTCTTATTC R: CGTTCTTCCGCTTCTCTCTTAC	75
	Zeb2	F: GAGAAAGTACCAGCGGAAACA R: AGGAGTCGGAGTCTGTCATATC	98
	Vimentin	F: TCTACGAGGAGGAGATGCGG R: GGTCAAGACGTGCCAGAGAC	213
Apoptosis genes	BCL2	F: ACTGGAGAGTGCTGAAGATTG R: CAGCATGATCCTCTGTCAAGT	88
	BCL2L12	F: ACCCGTGGACTTGAACTTG R: CTGAGTGGAATAGGAAGACTTGG	116
	Fas	F: ATTCTGCCATAAGCCCTGTC R: CTGTGTACTCCTTCCCTTCTTG	107
	Caspase3	F: CCTACAGCCCATTTCTCCATAC R: GCCTCACCACCTTTAGAACAT	125
	BAX	F: ATCATGGGCTGGACATTGG R: TGGAGACAGGGACATCAGT	117
ABC transporter genes	ABCG2	F: TTCCACGATATGGATTTACGG R: GTTTCCTGTTGCATTGAGTCC	83
	ABCB1	F: GTTCAGGTGGCTCTGGATAAG R: AGCGATGACGTCAGCATTAC	93
	ABCC1	F: CGCCTTCGCTGAGTTCCT R: TGCGGTGCTGTTGTGGTG	217
Telomerase reverse transcriptase gene	hTERT	F: CGGAAGAGTGTCTGGAGCAA R: GGATGAAGCGGAGTCTGGA	148

### Telomerase Activity by Telomeric Repeat Amplification Protocol (TRAP) Assay

Telomerase activity was determined by the TRAPEZE® XL Telomerase Detection Kit (Millipore, USA) according to the manufacturer's instructions. In brief, cells were cultured in a monolayer or spheroid condition. 10^6^ cells from two populations were lysed in 200 μL of cold CHAPS lysis buffer, incubated on ice and then centrifuged at 12,000 × g for 20 min at 4°C. A total of 2 μL cell extract was used to detect telomerase activity using the PCR system. In this reaction, heat-treated cell extract was used as the negative control and the control cell pellet provided in the kit was used as a positive control. Different dilutions from standard samples were prepared which included 1:02, 1:04, 1:09, and 1:19. Preincubation at 30°C for 30 min was carried out and then PCR amplification was performed as follows: initial incubation at 94°C for 30 s, 59°C for 30 s, and 72°C for 1 min for 36 cycles followed by a 72°C for 3 min extension step and then at 55°C for 25 min. The PCR products were electrophoresed on 10% polyacrylamide gel electrophoresis, stained with ethidium bromide. Subsequently, the yield of the PCR reaction was added in equal amounts into a 96-well plate and all steps were done according to the protocol. The absorbance of the samples was measured at 620 nm (27).

### Cell Culture and Treatment of PCs and SDCs With MST-312 as a Telomerase Inhibitor

An equal number of cells from PCs and SDCs were seeded in 200 μl DMEM with 10% FBS and in 200 μl serum-free medium using 96-well culture plates and ultra-low-attachment 96-well round bottom plates (all, Corning, Costar, USA), respectively, in triplicate. EGF and bFGF were added every other day for the forming of SDCs. MST-312 (EMD, Millipore, USA) is a synthetic compound that has been shown to be a potent telomerase inhibitor (28). MST-312 powder was reconstituted to 5 mg/mL in dimethyl sulfoxide, and further diluted to the desired concentration. After growing the PCs and SDCs, MST-312 was added at concentrations of 1 and 10 μM and incubated for 24, 48, and 72 h (29).

### Cell Viability Measured by CellTiter-Glo® Luminescent Assay

The CellTiter-Glo® Luminescent Cell Viability Assay (Promega, USA) is a homogeneous method to determine the number of viable cells in culture based on quantitation of ATP was used following the manufacturer's instruction. Briefly, the assay buffer and substrate were equilibrated at room temperature, and the buffer was transferred to substrate and gently mixed. The cell plates were equilibrated for 30 min at room temperature. The SDCs were transferred to white opaque walled 96-well plates followed by the addition of 100 μl of the assay reagent and incubation for 15 min on an orbital shaker to induce cell lysis. The plates were incubated at room temperature for 10 min to stabilize the signal, and luminescence was then read (Modulus™ II, BioSystems, USA).

### Statistical Analyses

All experiments were performed in triplicate. The data are expressed as the mean ± standard deviation (SD). Comparisons between groups were performed using a two-tailed paired Student *t-*test, and a *P*-value of < 0.05 was considered statistically significant.

## Results

### Spheroid-Derived From Parental ACHN Cells Formed Stable Spheroid Compared to the Other Cell Lines

**Figure 1:**
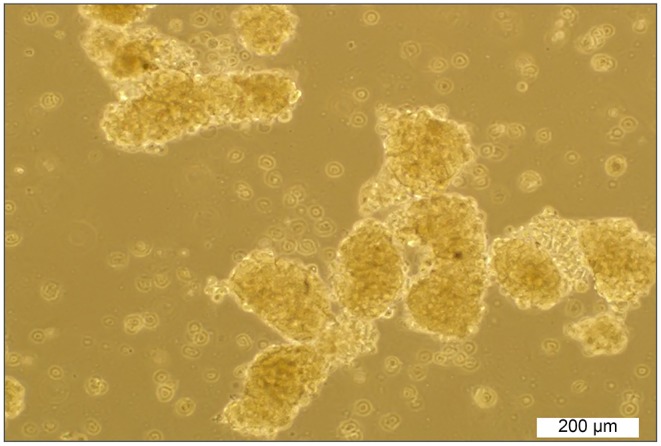
Sphere formation by ACHN cells. Spheroids derived from parental ACHN cells cultured in anchorage-independent conditions formed typical spheroids in the presence of growth factors at the second passage.

Initially, the sphere-forming ability in a panel of clear cell RCC cell lines, including ACHN, 786-O, and Caki-1, was tested. The SDCs from parental ACHN cells formed free-floating cellular aggregates and formed compact spherical shapes in serum-free medium in the presence of 20 ng/ml bFGF and 10 ng/ml EGF after 10–12 days (Figure 1). The SDCs of ACHN were also capable of being sub-culture. In contrast, SDCs from the other cell lines of RCC could not be sub-cultured. The second generation of SDCs from the ACHN cell line were used in the following experiment.

### SDCs Exhibited a Higher Clonogenicity, Larger Size of Colony, and Higher Sphere Forming Ability Compared With PCs

**Figure 2:**
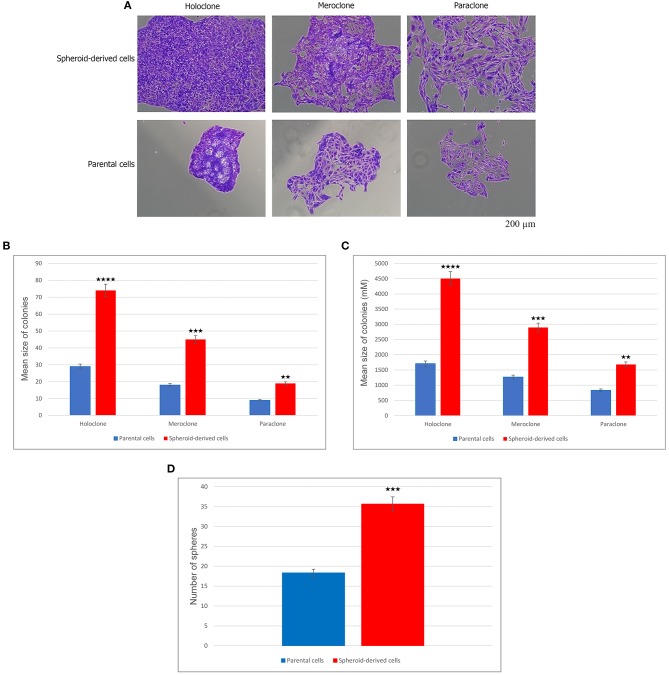
Clonogenicity and sphere-forming potential of parental cells (PCs) and spheroid-derived cells (SDCs). **(A)** Three types of colonies termed holo, mero, and paraclones were identified during the colony-formation assay in both PCs and SDCs. **(B)** The potential of clonogenicity was significantly higher in SDCs than in PCs. **(C)** The size of colonies was significantly larger in SDCs. **(D)** Results show a higher self-renewing capacity by SDCs compared to PCs. The colony number was counted under a dissection microscope. Data are represented as mean ± SD (*n* = 3 each). **P < 0.01, ***P < 0.001, and ****P < 0.0001.

To explore the CSC properties of SDCs, their clonogenicity, size of colony, and sphere-forming ability were compared to PCs. Three distinct types of colonies, including holoclone, meroclone, and paraclone, arose from each of the two cell populations. Holoclones were large with smooth edges and consisted of a homogenous population of small compact cells. Meroclones were smaller, irregular in shape, and made up of a mixture of small packed cells. Finally, paraclones were diffuse and comprised mainly of loosely packed cells (Figure 2A). Numbers of colonies were generated, and their mean colony size is shown in Table 2. Our findings have shown that there are statistically significant differences in colony-forming ability between PCs and SDCs with respect to the number of colonies and mean colony size (Figures 2B,C). In addition, PCs generated 18.3 ± 4.2 spheroids and SDCs 35.7 ± 3.1, a significantly higher sphere-forming ability than PCs (*P* < 0.001) (Figure 2D).

**Table 2:** Comparison of clonogenicity and size of colony in parental cells (PCs) compared to spheroid-derived cells (SDCs).

**Colony types**	**Number of cells**		***P*-value**	**Mean colony size (μm)**		***P*-value**
	**PCs**	**SDCs**		**PCs**	**SDCs**	
Holoclone	28.7 ± 10.1	74.0 ± 19.7	*<0.0001*	1708.8 ± 100	4508.3 ± 153	*<0.0001*
Meroclone	17.3 ± 3.5	45.3 ± 6.1	*<0.001*	1267.9 ± 46	2893.7 ± 3.9	*<0.001*
Paraclone	9.3 ± 1.5	19.7 ± 2.1	*<0.01*	835.0 ± 57	1680.8 ± 18	*<0.01*

*The values in italics are statistically significant*.

### SDCs Exhibited Higher Expression of Putative Cancer Stem Cell Markers Compared to PCs

**Figure 3:**
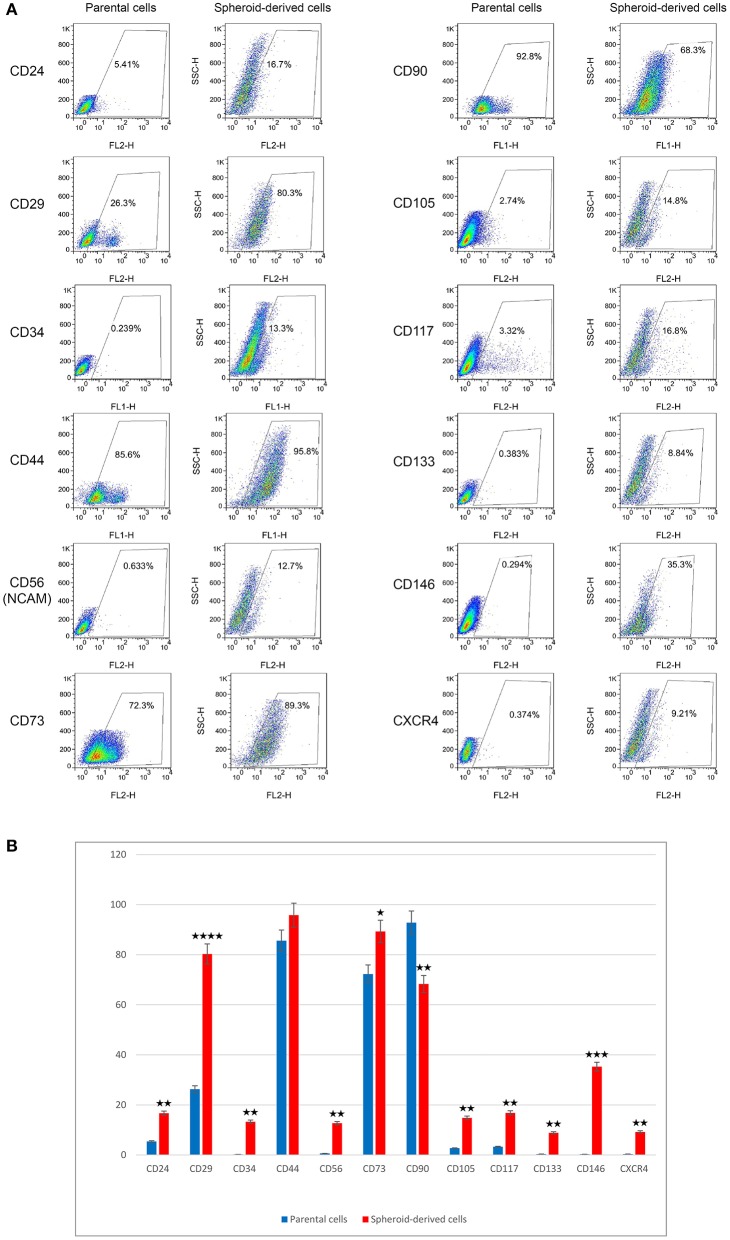
Expression levels of putative CSC markers in parental cells (PCs) and spheroid-derived cells (SDCs) using flow cytometry. The results of **(A,B)** show higher expression in CD29 and reduced expression of CD90 in SDCs compared with PCs. Other markers, except CD44, showed higher expression in SDCs than in PCs. Data are represented as mean ± SD (*n* = 3 each). *P < 0.05, **P < 0.01, ***P < 0.001, and ****P < 0.0001.

We next evaluated the expression of putative CSC markers in PCs and SDCs using flow cytometry. In this study, we found a much higher expression of CD29 in SDCs (80.3%) compared with PCs (26.3%). In contrast, SDCs showed less expression of CD90 (68.3%) than PCs (92.8%). In addition, the expression of CD24 (5.41%), CD34 (0.23%), CD56 (NCAM) (0.63%), CD73 (72.3%), CD105 (2.74%), CD117 (3.32%), CD133 (0.38%), CD146 (0.29%), and CXCR4 (0.37%) in PCs was lower than SDCs (16.7, 13.3, 12.7, 89.3, 14.8, 16.8, 8.84, 35.3, and 9.21%, respectively). There was no significant difference in expression of CD44 between PCs (85.6%) and SDCs (95.8%) (Figures 3A,B).

### SDCs Showed Increased Invasion Potential Compared to the PCs

**Figure 4:**
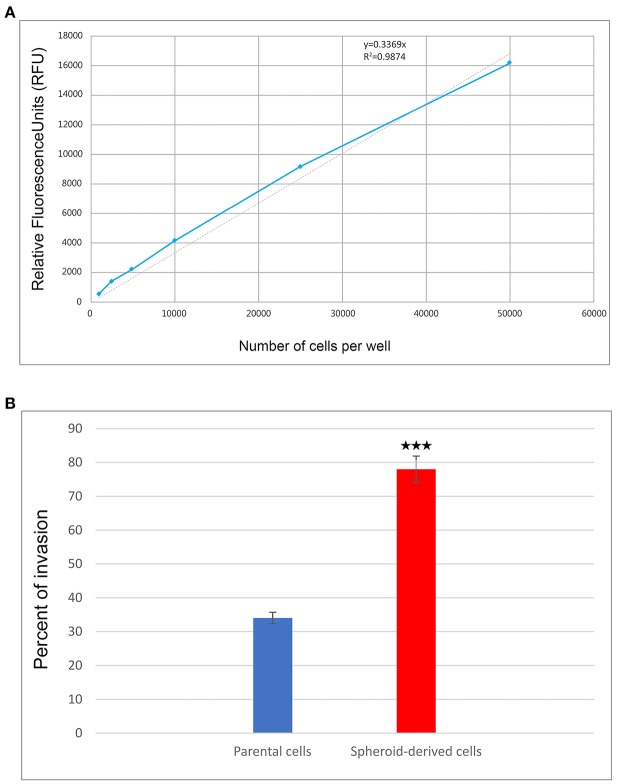
The results of cell invasion by parental cells (PCs) and spheroid-derived cells (SDCs). **(A)** The graph shows a cell invasion standard curve. ACHN cells were harvested, diluted, incubated for 1 hour with Calcein-AM, and assayed for fluorescence. The trend line and line equation were included on the graph. **(B)** The final results of cell invasion assay showed that SDCs are significantly more invasive (2.3-fold) than PCs. Data are represented as mean ± SD (*n* = 3 each). ***P < 0.001.

We performed a cell invasion assay to evaluate the invasive properties of PCs and SDCs. Using the manufacturer's recommendations, a standard curve was generated, and a best-fit line was plotted from the point zero based on the linear equation and regression coefficient or R2 (Coefficient of determination) (Table 3, Figure 4A). The average of all wells

**Table 3:** The data used for plotting the standard curve.

**Cells/well**	Average RFU^*^	**Background**	**Corrected RFU**
50,000	15,663	−236	15,427
25,000	8,434	−236	8,198
10,000	4,634	−236	4,398
5,000	2,448	−236	2,212
2,500	1,412	−236	1,176
1,000	722	−236	486
0	236		

* *RFU indicates Relative Fluorescence Units*.

for each condition was calculated and the background was subtracted from averages. The trend line equation was used to determine the number of cells present in each well; for the equation, y = mx + b, y value was replaced with relative fluorescence units (RFU) and solved for X (number of cells per well). The number of invaded cells was divided by the number of starting cells to determine percent invasion. The results showed a higher invasive potential in SDCs compared to the PCs (*P* < 0.001) (Table 4, Figure 4B).

**Table 4:** The final results of the cell invasion assay.

**Cells**	**Parental cells**		**Spheroid-derived cells**	
**Media**	**DMEM** **(Serum free)**	**10% FBS**	**DMEM** **(Serum free)**	**10% FBS**
Average of cells in each well	242	5,889	3,401	13,138
Standard deviation (SD)	5.50	154.56	264.97	218.22
Corrected RFU^*^	4	5,651	3,163	12,887
Number of invaded cells	12	17,126	9,884	39,052
Invasion%	0%	34%	19%	78%

* *RFU indicates Relative Fluorescence Units*.

### SDCs Exhibited Increased Tumorigenic Ability *in vivo*

**Figure 5:**
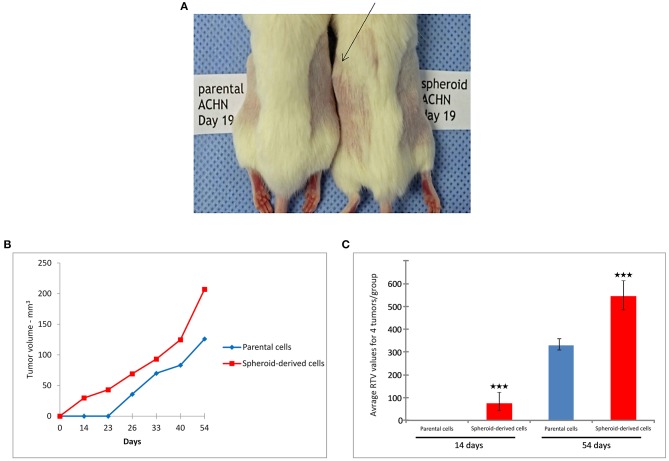
Tumorigenicity in non-obese diabetic/severe combined immunodeficiency (NOD/SCID) mice. **(A)** Tumors derived from spheroid-derived cells (SDCs) were observed in NOD/SCID mice 19 days after injection, whereas no tumors were apparent in mice xenografted with parental cells (PCs) over the same time frame. **(B)** Tumor volume curves and **(C)** relative tumor volume (RTV) of PCs and SDCs. We observed a significantly earlier tumor onset and higher tumor volume in mice xenografted with SDCs compared to PCs. (Number of mice = 4). Data are represented as mean ± SD (*n* = 3 each). ***P < 0.001.

The tumorigenic potential of PCs and SDCs in a xenograft model was also examined. The injection of 1 × 10^3^ or 1 × 10^4^ cells was unable to generate tumors in NOD/SCID mice, while 1 × 10^5^ cells generated tumors in mice. Tumors derived from SDCs were observed on day 14 while tumors from PCs were not visible until day 26 in NOD/SCID mice (*P* < 0.001). In addition, tumors of the SDC xenografts were significantly larger than the PC xenografts (*P* < 0.001) (Figures 5A–C).

### Spheroid-Derived Xenografts Show Significantly Increased Levels of Stemness and EMT Genes and Exhibited Higher Expression of Stemness Markers

**Figure 6:**
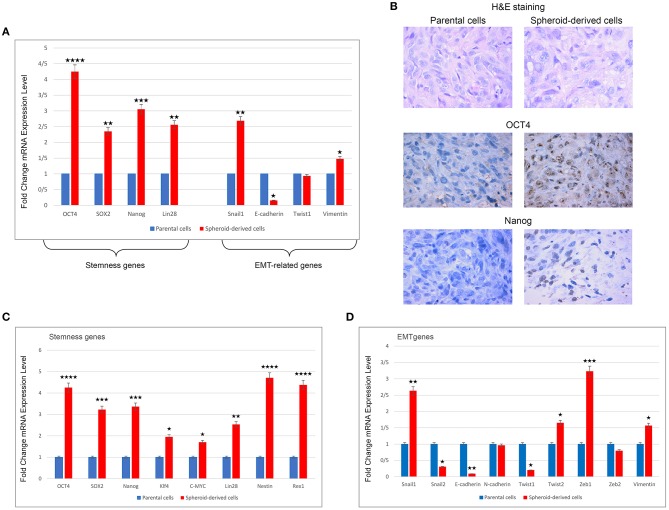
Expression levels of stemness and EMT genes using qRT-PCR, H&E, and immunohistochemical staining in xenograft tumors derived from parental cells (PCs) and spheroid-derived cells (SDCs) and expression levels of stemness and EMT genes in PCs of ACHN cell line and their SDCs by qRT-PCR. **(A)** Expression of all stemness genes and some EMT-related genes were higher in SDCs from xenograft tumors compared to PCs. **(B)** Results of H&E staining showed a clear cell RCC morphology. Immunohistochemical staining indicated increased expression of OCT4 and Nanog in tumors from SDCs compared to PCs. **(C)** SDCs showed significantly higher mRNA levels of all stemness genes compared to PCs. **(D)** In EMT-related genes, significantly higher levels of Snail1, Twist2, Zeb1, and Vimentin mRNA and significantly lower levels of Snail2, E-cadherin, and Twist1 mRNA were observed in SDCs compared to PCs. There were no significant differences in mRNA expressions of N-cadherin and Zeb2 in the two cell populations. Data are represented as mean ± SD (*n* = 3 each). *P < 0.05, **P < 0.01, ***P < 0.001, and ****P < 0.0001.

qRT-PCR analysis was performed to compare the expression of a panel of stemness and EMT-related genes in xenograft tumors from PCs and SDCs. A higher expression of OCT4 (*P* < 0.0001), Nanog (*P* < 0.001), SOX2, and Lin28 (all *P* < 0.01) was found in tumors derived from SDCs compared to tumors from PCs. In addition, EMT-related genes, Snail1 (*P* < 0.01), and Vimentin (*P* < 0.05) were expressed at higher levels in tumors from SDCs compared to tumors from PCs (Figure 6A). H&E staining of xenografts confirmed that the tumors that formed were typical human clear cell RCC. IHC indicated that the expression of the stemness markers, OCT4, and Nanog were higher in xenograft tumors derived from SDCs than xenograft tumors from PCs (Figure 6B).

### qRT-PCR Analysis of PCs and SDCs

**Figure 7:**
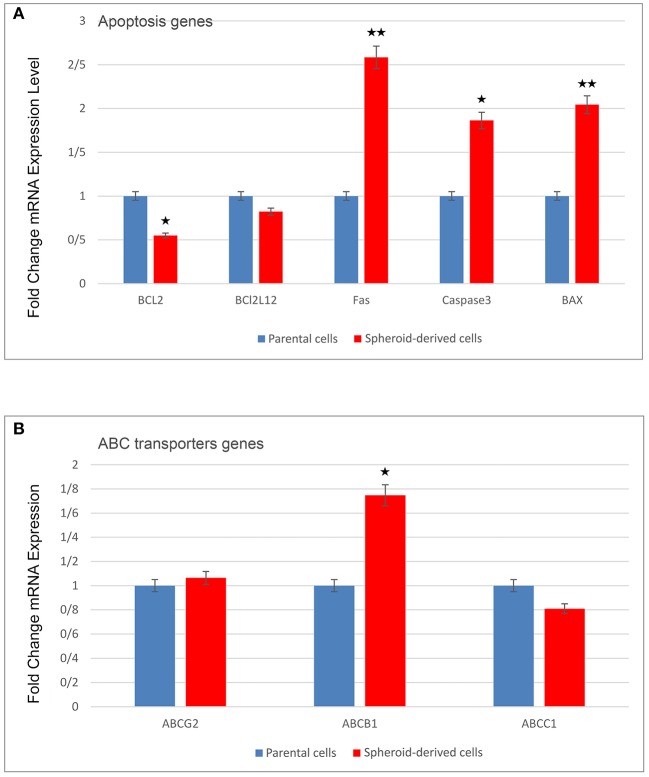
A comparison of pro- and anti-apoptotic genes as well as ABC transporters genes expression in parental cells (PCs) and spheroid-derived cells (SDCs) by qRT-PCR. **(A)** Anti-apoptotic gene BCL2 was lower and pro-apoptotic genes, including Fas and casapase-3, and Bax were higher in SDCs compered to PCs. **(B)** ABCB1 was expressed higher in SDCs than in PCs among ABC transporter genes examined. Data are represented as mean ± SD (*n* = 3 each). *P < 0.05, **P < 0.01.

**Figure 8:**
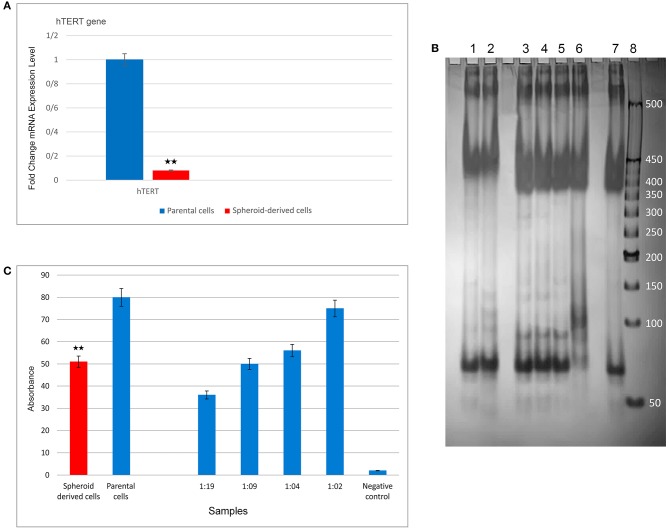
Expression levels of hTERT and telomerase activity using qRT-PCR and TRAP assay (PCR and ELISA) in parental cells (PCs) and spheroid-derived cells (SDCs), respectively. **(A)** Expression of hTERT was lower in SDCs than PCs. **(B)** PCR products were electrophoresed on a 10% polyacrylamide gel to evaluate the telomerase activity. Lane 1: SDCs, lane 2: PCs, lanes 3– 6: positive controls diluted: 1:19, 1: 09, 1: 04, and 1: 02, respectively, lane 7: heat-treated cell extract (negative control), and lane 8: 50 bp DNA Ladder. Positive controls (lanes 3–6) exhibited a strong band 90 bp indicative of telomerase activity, and the negative control (lane 7) did not exhibit this telomerase-specific band. The telomerase activity in lane 2, containing PCs, demonstrated more strong banding compared to the SDCs in lane 1. **(C)** The results of telomerase activity using ELISA showed reduced telomerase activity in SDCs compared to the PCs. Also, we observed that increased expression of telomerase activity in control samples from 1:19 to 1:02. Data are represented as mean ± SD (*n* = 3 each). **P < 0.01.

qRT-PCR was carried out to compare the expression of a panel of genes involved in the CSC phenotype. As expected, SDCs expressed significantly higher levels of all stemness genes, including OCT4 (*P* < 0.0001), SOX2 (*P* < 0.001), Nanog (*P* < 0.001), Klf4 (*P* < 0.05), C-MYC (*P* < 0.05), Lin28 (*P* < 0.01), Nestin (*P* < 0.0001), and Rex1 (*P* < 0.0001) (Figure 6C). A significantly higher level of Snail1 (*P* < 0.01), Twist 2 (*P* < 0.05), Zeb1 (*P* < 0.001), and Vimentin (*P* < 0.05) and a significantly lower level of Snail2 (*P* < 0.05), Twist 1 (*P* < 0.05), and E-cadherin (*P* < 0.01) were also observed in SDCs compared to PCs (Figure 6D). A significantly higher level of pro-apoptotic genes, Fas (*P* < 0.001), Caspase3 (*P* < 0.01), and BAX (*P* < 0.001), and a lower level of anti-apoptotic gene BCL2 (*P* < 0.05) was seen in SDCs compared to PCs (Figure 7A). Among ABC transporters genes, ABCB1 (*P* < 0.05) was the only gene expressed at significantly higher levels in SDCs than PCs (Figure 7B). In addition, SDCs exhibited decreased hTERT expression (*P* < 0.01; Figure 8A) compared to PCs.

### SDCs Presented Lower Telomerase Activity Compared to PCs

The TRAP assay (based on PCR and ELISA) was employed to compare telomerase activity between the two cell populations. In this test, heat-treated cell extract was used as the negative control and the control cell pellet provided in the kit was applied as a positive control. Different dilutions from standard samples were prepared, including 1:02, 1:04, 1:09, and 1:19, and PCR amplification was then performed. The PCR products were electrophoresed on a 10% polyacrylamide gel. Subsequently, the yield of the PCR reaction was added in equal amounts into a 96-well plate and all steps were done according to the protocol. In this study, positive control samples exhibited a strong band 90 bp, indicating telomerase activity, and negative control did not exhibit this specific band. The lane containing PCs, exhibited more intense telomerase banding compared to the SDCs indicating reduced telomerase activity in SDCs compared to PCs (Figure 8B). The results of telomerase activity using ELISA also showed that SDCs had significantly reduced telomerase activity compared to PCs (*P* < 0.01; Figure 8C).

### MST-312 Acts Preferentially on SDCs Compared to the PCs After 72 h

**Figure 9:**
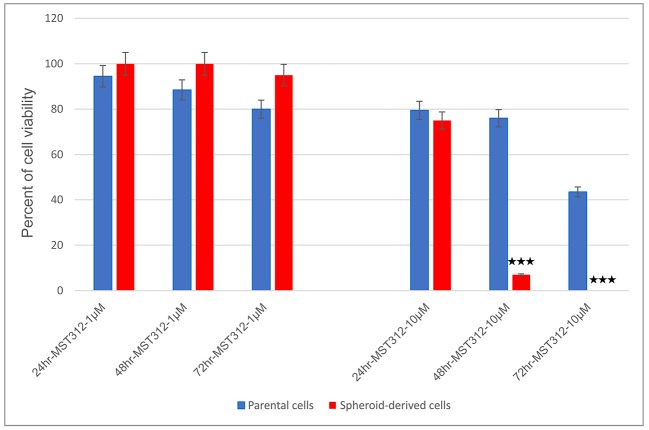
Telomerase inhibition with MST-312 in parental cells (PCs) and spheroid-derived cells (SDCs). The PCs and SDCs were treated with MST-312 at 1 μM (left) and 10 μM (right) for 24, 48, and 72 h. After 48 and 72 h of 10 μM MST-312 treatment, there was a greater decrease in cell viability of SDCs compared to PCs. Data are represented as mean ± SD (*n* = 3 each). ***P < 0.001.

To identify whether MST-312, a telomerase inhibitor, acts preferentially on SDCs over PCs, the two populations were treated with MST-312 at concentrations of 1 μM and 10 μM for 24, 48, and 72 h and assessed for cell viability. At 10 μM, SDCs were profoundly more sensitive to MST-312 than PCs; the percentage of viable PCs after 48 and 72 h was 76 and 43.5%, respectively, whereas the percentage of viable SDCs over the same period of time was 7 and 0%, respectively (Figure 9).

## Discussion

Current therapies for RCC are often met with therapeutic failure, recurrence, and metastasis. Tyrosine-kinase inhibitors (TKIs) were introduced as a breakthrough in the treatment of metastatic RCC (30, 31); however, most metastatic RCCs develop drug resistance to TKIs and eventually progress (32). In addition, efficient immunotherapy for RCC is limited (33). One explanation for the development of therapy resistance is the CSC model wherein a small population of the cells within the tumor, known as CSCs, are largely responsible for cancer relapse (34). As such, studies aiming to improve our understanding of the biological characteristics of CSCs might facilitate the development of novel therapeutic strategies that target this cell population. Moreover, the unique role that telomerase fills in conferring normal stem cells with immortality is well-established and brings about the possibility that it has a similar role in CSCs (16). Since there are currently no universal markers for the isolation and identification of CSCs, their characterization is mainly based on functional studies (35). In the current work, SDCs were enriched and expanded using a sphere culture system in non-adherent conditions. Among all cell lines tested, only metastatic ACHN cells were able to generate SDCs in the second passage. Our findings showed that SDCs displayed a significantly higher clonogenicity, larger colony size, and higher sphere-forming ability than the PCs, all considered to be CSC features (6). Flow cytometric analysis of a panel of putative CSC markers indicated increased expression of putative CSC markers in SDCs compared to PCs. These data demonstrated that SDCs from the ACHN cell line can be considered as a cancer stem-like cell population.

Increased invasion and drug resistance by CSCs presents major challenges in the management of RCC patients (6), and we therefore the invasiveness of PCs and SDCs. Results showed that cancer cells grown as SDCs have a higher invasive potential compared to PCs. Previous work in breast cancer also showed that breast CSCs are more invasive than non-CSCs (36). In our study, we observed a significantly early tumor formation and higher tumor volume in NOD/SCID mice injected with SDCs compared to mice injected with PCs. Increased expression of stemness and mesenchymal genes and reduced expression of E-cadherin was observed in our SDCs, suggesting CSC properties. According to the CSC theory, this type of cell is capable of self-renewal and producing larger tumors in NOD/SCID mice compared to PCs (37, 38).

We analyzed the expression profiles of eight stemness genes which showed a higher expression in SDCs compared to PCs. The factors OCT4, Nanog, SOX2, Klf4, C-MYC, and Lin28 play crucial roles in cancer progression. Several groups have demonstrated that inhibition or knockdown of these genes results in the reduction of CSC characteristics (39, 40). This study is the first to investigate the expression of Nestin and Rex1 in the ACHN PCs and their SDCs. Some experimental findings suggest that Nestin expression is related to the capacity for self-renewal (41). Taken together, our results suggest that targeting of these stemness genes may reduce or perhaps completely neutralize the renal CSCs.

Recently, CSCs have been shown to play an important role in EMT (42). Therefore, in the next set of experiments, we examined the expression of several EMT-related genes. Snail1 and Snail2, Zn-finger transcription factors, suppress the expression of E-cadherin and can activate EMT (43). In the present study, increased expression of snail1 and decreased expression of E-cadherin were observed in SDCs compared to PCs. The reduced expression of E-cadherin in an epithelial cell has been considered a hallmark of EMT (43). It has been shown that loss of Snail1 in mesenchymal cells can cause downregulation of Nanog and loss of self-renewal characteristics *in vitro* (42). Expression of Twist1 and Twist2 in human solid tumors has been associated with tumor progression (44). In our study we found increased expression of Twist2 and reduced expression of Twist1 in SDCs, which is in contrast with previous study on Twist1 (13). This might be related to low expression of Snail2, as a previous study showed that Snail2 is an essential mediator for induction of Twist1 to promote EMT in culture (43). We found a significant 3.2-fold increase of Zeb1 and higher expression of Vimentin in SDCs compared to PCs. Given the strong links between EMT and invasiveness and the central role of invasiveness in metastasis, our findings suggest that targeting genes upregulated in our SDC population, including Zeb1, Snail1, Twist2, and Vimentin could offer new therapeutic approaches for RCC.

Increasing data suggests that a CSC population leads to treatment resistance by mechanisms that include induction of anti-apoptotic genes and a number of membrane transporters, such as ABC transporters (45, 46). As such, we examined a panel of genes involved in apoptosis and ABC transporters. Our real-time PCR assays showed that pro-apoptotic genes, including Fas, casapase-3, and Bax, were highly expressed in SDCs compared to the PCs, and the anti-apoptotic gene, Bcl-2, was decreased in SDCs. These findings suggest that our SDCs not only have anti-apoptotic programs in effect, but also that pro-apoptotic programs engaged, indicating that this cell population might employ another mechanism that does not involve apoptosis to confer drug resistance. We also found increased expression of ABCB1 in SDCs compared to the PCs. Our findings suggest that drug resistance in renal CSCs may be due to the increased expression of ABC transporters genes, such as ABCB1, rather than suppression of apoptotic genes. This study has a limitation. The transcription data is useful for identifying potential candidates for follow-up work at the protein level. Due to translational modulation and post-translational modification, protein levels do not necessarily reflect gene expression levels, especially for enzymes with post-translational modification and structural or configuration changes at the catalytic core (47). Therefore, to confirm our findings, protein levels should be measured.

The results of our hTERT gene expression analysis and evaluation of telomerase activity showed for the first time that telomerase is downregulated in renal SDCs compared to PCs. Our findings are in agreement with a previous study on brain CSCs, which found that reduced expression of hTERT and telomerase activity in brain CSCs compared to PCs (48). Moreover, hTERT has been demonstrated to be downregulated in the CD34+ haematopoietic stem cells of patients with chronic myeloid leukemia. This could be correlated to the genomic instability, suggesting the progressive decrease of hTERT expression with disease evolution (49). Previous studies have shown that downregulation of telomerase activity may be due to deacetylation of histones H3 and H4 at the hTERT promoter and deacetylation of histone H3 at the hTR promoter during differentiation. Histone modification has been shown to be an important means by which telomerase expression is regulated (50). A previous study found that telomerase activity is different between the CSCs and PCs among multiple myeloma patients and from cell line to cell line (51). Another study demonstrated that breast CSCs have lower telomerase activity than PCs, while pancreatic CSCs exhibited higher telomerase activity compared to PCs (52). In addition, Serrano et al. (53) observed no statistically significant difference in the telomerase activity between lung CSCs and PCs. Collectively, these studies suggest that telomerase activity enzyme is not universally utilized by CSCs.

It has been suggested that CSCs could be sensitive to targeted therapies against telomerase (54). MST-312 is a derivative of a component of green tea and a novel telomerase inhibitor with potential therapeutic activity (55, 56). MST-312 does not inhibit the growth of normal cells but effectively inhibits metastatic cancer cells (56). Recently, Ghasemimehr et al. reported that MST-312 did not show any cytotoxic and apoptotic effects on normal human peripheral blood mononuclear cells (PBMCs), suggesting selectivity for tumor cells (57). There are multiple pathways that can be inhibited by MST-312. Previous studies have shown that MST-312 acts through two different mechanisms depending on the time of exposure. Short-term administration of the inhibitor results in induced G2/M cell cycle arrest and acute ATM-pathway-dependent DNA damage and reduced cell viability. This effect, which occurs within 72 h of treatment, is not mediated by telomere erosion. Long-term effects of this inhibitor after 1.5 months of exposure leads to a significant telomere shortening in SDCs compared to the PCs (53, 58). Another study showed that MST-312 induces G2/M cell cycle arrest and apoptosis in acute promyelocytic leukemia (APL) cells through the inhibition of telomerase activity and suppression of the nuclear factor-κB (NF-κB) signaling pathway (59).

In the current study, MST-312 was used as a short-term telomerase inhibitor in PCs and SDCs. We are the first to report that telomerase inhibition leads to decreased viability of PCs as well as SDCs. Our findings showed that, at 10 μM, MST-312 drastically reduced the percentage of viable PCs and SDCs to 76 and 7%, respectively, after 48 h of exposure. Furthermore, MST-312 was able to eliminate 56.5% of PCs and 100% of SDCs after 72 h of treatment. MST312 has been studied in different cancer cell lines, including brain cancer, non-small cell lung cancer (NSCLC), and breast cancer (53, 55, 56). Gurung et al. showed that treatment with MST-312 decreased brain tumor cell viability (55). The results of Serrano et al. showed that MST-312 reduces the number of lung CSCs (53). In another study in breast cancer, MST-312 decreased telomerase activity and induced telomere dysfunction and growth arrest in breast cancer cells (56). The results of our study indicate that SDCs were considerably more sensitive to MST-312 than PCs. It is possible that downregulated telomerase in CSCs leads to shorter telomere length in the SDCs rendering them more sensitive to telomerase inhibitor. Previous investigations have shown that in the absence of active telomerase, telomeres will shorten at each cell division and when the telomeres reach a critically short length, chromosomal instability, cell senescence, and apoptosis are triggered. Also, there is a meaningful relationship between the shortening of telomeric length and chromosomal instability (60). As we accrue more information in different renal cancer cell lines on the roles of the stem-like and non-stem-like populations in cancer development, the possibility of whether or not we can use MST-312 as a complementary telomerase-targeted treatment modality in RCC will be realized. However, this hypothesis should be confirmed in future studies using a larger variety of cell lines with different concentration of MST-312.

In conclusion, renal SDCs exhibited the characteristics of CSCs, including high clonogenicity, enhanced colony-forming ability, increased expression of putative CSCs, increased invasiveness, high tumorigenicity in xenograft models, and increased expression of both stemness and EMT-related genes. As such, sphere culture systems may be a useful and effective preclinical model for investigating renal cancer, especially for testing targeted therapies against CSCs. Our gene expressions analyses revealed a number of prospective targets that relate to stemness and EMT, including Zeb1, Snail1, Twist2, Vimentin, as well as the ABC transporters gene, ABCB1. In addition, our findings showed that SDCs express pro-apoptotic factors, suggesting drug resistance in renal CSCs may be due to increased expression of ABC transporters genes, such as ABCB1, rather than suppression of apoptotic genes. Our study reported the reduced expression of hTERT and telomerase activity in CSCs enriched from an RCC cell line indicating that expression of hTERT and telomerase activity are not always higher in CSCs. We demonstrate here for the first time that the novel telomerase inhibitor MST-312 inhibits cells of SDCs more strongly than PCs and may therefore be useful as a complementary targeted therapy against renal CSCs. Further studies should be carried out to confirm and expand the findings of this study.

## Data Availability Statement

All datasets generated for this study are included in the article.

## Ethics Statement

This study didn't involve human subjects. The *in vivo* experimental study was performed according to regulations of the Federation of European Laboratory Animals Science Association (FELASA), and all procedures were approved by the National Animal Research Authority.

## Author Contributions

LS, a Ph.D. student, performed all laboratory procedures, data analysis, and wrote the primary manuscript. They were supervised by ZM and MAs who designed this research study. MAb was a consultant on this project. AR contributed to most of the experiments in the lab. RR helped during some experiments and made suggestions on the manuscript. KT contributed to editing the manuscript in native English and gave helpful comments on the whole manuscript. YA was the supervisor at the department of tumor biology in Norway. She helped us in all laboratory procedures, especially in animal experiments. ØF and GM helped during discussions and made suggestions on the manuscript. All authors involved in review of the manuscript and approved the final version of this manuscript.

### Conflict of Interest

The authors declare that the research was conducted in the absence of any commercial or financial relationships that could be construed as a potential conflict of interest.
